# Autoimmune Hepatitis with Elevated Serum IgG4 Levels Have a High Prevalence of Cirrhosis at Diagnosis

**DOI:** 10.1155/2021/6692511

**Published:** 2021-01-04

**Authors:** Mei Xue, Yi Shen, Xiaoli Fan, Mengyi Shen, Li Yang

**Affiliations:** Department of Gastroenterology & Hepatology, West China Hospital, Sichuan University and Sichuan University-University of Oxford Huaxi Joint Centre for Gastrointestinal Cancer, Chengdu, Sichuan 610041, China

## Abstract

**Background:**

Some autoimmune hepatitis (AIH) patients have elevated serum IgG4 levels, and the clinical characteristics of such patients are currently incompletely characterized.

**Aim:**

To analyze the clinical features and possible pathogenesis of AIH with elevated serum IgG4 levels.

**Methods:**

According to their serum IgG4 value, patients were divided into elevated IgG4 (IgG4 > 1.35 g/l) and normal IgG4 (IgG4 ≤ 1.35 g/l) groups.

**Results:**

Among the 152 patients included in this study, those in the elevated IgG4 group had the following characteristics: older onset age (56 ± 11.43 years vs. 49.49 ± 13.04 years, *P*=0.005), higher proportion of males (34.15% vs. 12.61%, *P*=0.002), higher prevalence of cirrhosis (56.10% vs. 36.04%, *P*=0.026), lower prevalence of extrahepatic autoimmune diseases (9.76% vs. 27.3%, *P*=0.023), and higher levels of IL-17 and IL-22 (*P* < 0.05). Logistic regression analysis results showed that elevated serum IgG4 levels and male sex were risk factors for AIH cirrhosis (male: odds ratio (OR) = 4.293, 95% confidence interval (CI): 1.592–11.575, *P*=0.004; and elevated serum IgG4: OR = 2.566, 95% CI: 1.065–6.187, *P*=0.036). No significant differences were found for the remission rate within 6 months between the two groups (69.70% vs. 76.14%, *P*=0.470).

**Conclusion:**

The male proportion and cirrhosis prevalence were higher in AIH with elevated serum IgG4 levels at the time of diagnosis. Male sex and elevated serum IgG4 levels are independent risk factors for AIH cirrhosis, and TH17 cells are more likely involved in the pathogenesis of this type of AIH.

## 1. Introduction

Autoimmune hepatitis (AIH) is an immune-mediated inflammatory liver disease that is common in women and characterized by elevated serum IgG levels, presence of serum autoantibodies, and interfacial hepatitis in liver histology [1, 2]. Clinically, some AIH patients have elevated serum IgG4 levels but are not diagnosed with IgG4-related diseases (IgG4-RD) or IgG4-related autoimmune hepatitis (IgG4-related-AIH). Currently, there is a lack of research on serum IgG4 in AIH. Even in IgG4-RD, the role of serum IgG4 is still not fully understood [3, 4]. However, Abe [5] had reported that type 1 AIH patients with elevated serum IgG4 levels had a higher rate of cirrhosis progression than patients with low serum IgG4 levels (80.0% vs. 24.1%, *P* < 0.05), suggesting a potential correlation between the increase in serum IgG4 levels and the occurrence of cirrhosis. However, the sample size of this study was a small and the pathogenic mechanism of cirrhosis was not proposed.

Considering that studies on the clinical features and pathogenesis of AIH with elevated serum IgG4 levels are lacking, and some existing research [6, 7] have pointed out that the degree of TH17 cells infiltration into the AIH liver is related to the degrees of liver inflammation and fibrosis, so this study assessed the clinical features of AIH with elevated serum IgG4 levels and the possible role of TH17 cells in the pathogenesis of this disease.

## 2. Patients and Methods

### 2.1. Study Population

Our center has collected all patients considered AIH between June 20, 2014, and February 1, 2020, and these patients were included for research according to the following conditions: (1) the revised International Autoimmune Hepatitis Group (IAIHG) score [8] before treatment was ≥10; (2) serum IgG4 was detected; (3) age ≥18 years; (4) liver diseases caused by primary biliary cholangitis, primary sclerosing cholangitis (PSC), IgG4-RD, IgG4-related-AIH, alcoholic liver disease, drug-induced liver injury, viral hepatitis, and other causes were excluded. According to the detection results for serum IgG4 determined before treatment, the enrolled patients were divided into an elevated serum IgG4 group (serum IgG4 > 1.35 g/l) and a normal IgG4 group (serum IgG4 ≤ 1.35 g/l). All patients who received immunosuppressive treatment were examined at least every 3 to 6 months to monitor their condition. In special cases, a shorter interval of clinical follow-up was used according to the patient's condition.

In this study, cirrhosis was defined as 4^th^ stage of histological fibrosis or imaging cirrhosis.

Clinical manifestations included clinical symptoms (e.g., jaundice, fatigue, anorexia, and ascites), no clinical symptoms, extrahepatic autoimmune diseases (Hashimoto's thyroiditis had the strongest association, and patients also presented with Grave's disease, vitiligo, alopecia, rheumatoid arthritis, diabetes mellitus type 1, inflammatory bowel disease, psoriasis, systemic lupus erythematosus, Sjögren's syndrome, celiac disease, panniculitis, idiopathic thrombocytopenic purpura, polymyositis, hemolytic anemia, and uveitis), and liver cirrhosis. Treatment response was evaluated by remission, which was defined as normalization of transaminase levels after treatment [9, 10]. It has been reported [11] that patients with remission within 6 months after the initiation of immunosuppressive therapy have a better prognosis; thus, the 6^th^ month was selected as the time point for therapeutic response evaluation in this study.

### 2.2. Clinical Parameters (Laboratory, Histological, and Imaging)

Basic patient information, laboratory test results (including those for platelets, liver function, routine coagulation, immunoglobulin, and autoimmune antibodies), imaging examination results (including those for abdominal ultrasound, epigastric enhanced computed tomography, and magnetic resonance cholangiography), liver histological results (interface of hepatitis, lymphatic plasma cell infiltration, rose wreath sample change, cholestasis, liver fibrosis stage, grade of inflammation, IL-17 (1 : 400, 13082-1-AP, SanYing), and IL-22 (1 : 500, GB11259, Servicebio)), extrahepatic autoimmune disease (EAD) information, and treatment data were collected through the hospital electronic medical records system, WeChat, or telephone follow-up. Serum cytokines were detected with Luminex liquid-phase chips (AliveX Biotech, China). This study was reviewed and approved by the Ethics Committee of the West China Hospital, Sichuan University (no. 2013221). Informed consent was waived due to the retrospective nature of this study.

### 2.3. Statistical Analysis

Continuous variables conforming to a normal distribution were compared between groups by a *t*-test and are expressed as the mean ± standard deviation. Variables that did not conform to a normal distribution were compared between groups by the Mann–Whitney *U* test and are represented by the median (interquartile range). Categorical variables are expressed as the number of cases (percentage), and the chi-square test or Fisher's exact probability method was used for comparisons between groups. The variables with *P* < 0.1 after comparison with baseline data were included in a logistic regression analysis. SPSS v.25 was used for statistical analysis, and a *P* value < 0.05 was considered statistically significant.

## 3. Results

### 3.1. Comparison of Baseline Characteristics and Treatment Responses between Two Groups

A total of 152 patients including 41 in the elevated serum IgG4 group (A group) and 111 in the normal IgG4 group (B group) were enrolled in this study. The baseline information of the patients at the time of diagnosis is shown in Table 1. The age of onset was older (56 ± 11.43 years vs. 49.49 ± 13.04 years, *P*=0.005), the proportion of males was higher (34.15% vs. 12.61%, *P*=0.002), the prevalence of cirrhosis was higher (56.10% vs. 36.04%, *P*=0.026), and the prevalence of EAD was lower (9.76% vs. 27.03%, *P*=0.023) in the group with elevated serum IgG4 levels. In terms of laboratory tests, the IgG4, IgG, and GLB levels in the group with elevated serum IgG4 levels were higher than those in the normal group (*P* < 0.05); the IgM and albumin (ALB) levels in the elevated serum IgG4 group were lower than those in the normal IgG4 group (*P* < 0.05); and the total bilirubin (TB), alkaline phosphatase (ALP), gamma-glutamyl transferase (GGT) and prothrombin time (PT) showed trends of higher levels in the elevated serum IgG4 group than in the normal group (*P* was close to 0.05); however, bile duct lesions between the two groups showed no statistically significant difference (*P*=0.613). The transaminase level, platelet count, autoantibody positive rate, pretreatment score, the score of simplified criteria for the diagnosis of AIH, and histological inflammation were also not different between the two groups (*P* > 0.05). There were 88 and 33 patients in the normal and elevated serum IgG4 groups that received standard treatment, respectively; however, no differences were found for the remission rate within 6 months between the two groups (23 (69.70%) vs. 67 (76.14%), *P*=0.470).

Since Table 1 shows that the proportion of males and the rate of cirrhosis were higher in AIH patients with elevated serum IgG4 levels than in those with normal IgG4 levels, all patients were divided into groups according to sex for comparison. The main results for the two groups are summarized in Table 2. We concluded that the levels of IgG4 and the prevalence of cirrhosis in males were still higher than those in females (*P*=0.002 and 0.007, resp.), and there were no statistically significant differences in the degree of liver tissue inflammation or the age of onset between males and females (*P* > 0.05). The time interval from onset to diagnosis was also not significantly different (1 (1–3) year vs. 1.5 (1–3.75) years, *P*=0.688).

### 3.2. Risk Factor Analysis of Elevated Serum IgG4 Levels and AIH Cirrhosis

Table 3 shows that male sex was a risk factor for serum IgG4 level elevation (OR = 2.809, 95% CI: 1.034–7.629, *p*=0.043) and that EAD was a protective factor associated with no serum IgG4 level elevation (OR = 0.274, 95% CI: 0.078–0.967, *p*=0.044).  Table 4 shows that male sex and elevated serum IgG4 levels were independent risk factors for AIH cirrhosis (male: OR = 4.293, 95% CI: 1.592–11.575, *p*=0.004; elevated serum IgG4: OR = 2.566, 95% CI: 1.065–6.187, *P*=0.036).

### 3.3. Cytokine Detection in the Elevated Serum IgG4 Group and the Normal Group

**Figure 1:**
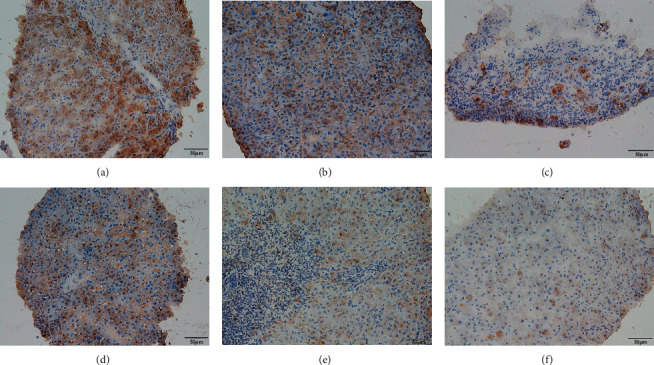
Comparison of IL-17 immunohistochemical stain of liver tissue. (a–c) Liver histology of serum IgG4 elevated group; (d–f) liver histology of serum IgG4 normal group.

**Figure 2:**
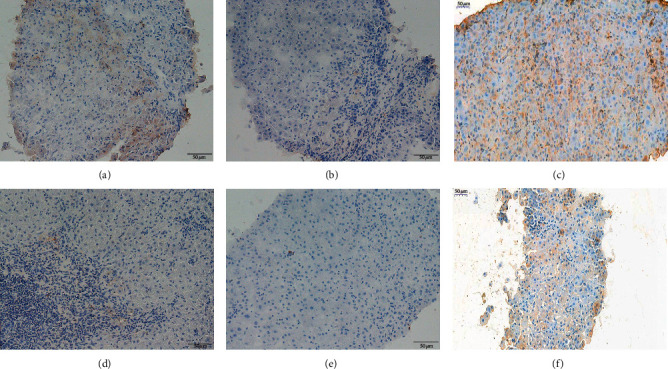
Comparison of IL-22 immunohistochemical stain of liver histology. (a–c) Liver histology of serum IgG4 elevated group; (d–f) liver histology of serum IgG4 normal group.

An existing study [6] suggests that interleukin (IL) 17 can stimulate a variety of types of nonparenchymal liver cells to secrete proinflammatory cytokines and chemokines to induce and promote liver inflammation in liver disease. IL-17 also promotes liver fibrosis by activating hepatic stellate cells [7]; therefore, we explored the differences in TH17-related cytokines between the elevated serum IgG4 group and the normal group. A total of 68 patients including 15 patients in the elevated serum IgG4 group and 53 patients in the normal IgG4 group were evaluated to detect TH17-related cytokines (IL-17A, IL-21, and IL-22). After comparison, the IL-17A and IL-22 levels in the elevated serum IgG4 group were higher than those in the normal group (Table 5, *P* < 0.05). And the immunohistochemical evaluation of IL-17 and IL-22 in liver histology also got similar results (Figures 1 and 2).

Interestingly, the onset age of AIH patients with elevated serum IgG4 levels was older. A previous study reported [13] that, compared with younger AIH patients, at the time of AIH diagnosis, elderly AIH patients were more often asymptomatic and were more frequently positive for antinuclear autoantibodies (ANA) and HLA-DR4. Among the extrahepatic manifestations, autoimmune thyroid disorders (AITD) were prevalent in the elderly group. Thus, our study compared the related clinical parameters between the elderly and younger patients, and the results are shown in Tables 6 and 7. We found that, compared with the younger group, the elderly group had a higher cirrhosis rate and IgG level, while the IgG4 level, proportion of male patients, aminotransferase level, ANA-positive rate, EAD, and remission rate did not differ between the groups. Unfortunately, due to our limited conditions, all patients were not tested for HLA-DR4. The prevalence of EAD in our study was 23.34%, and AITD were predominant (Table 7). There was no significant difference in the prevalence of AITD between the elderly and younger patients (8.57% vs. 8.55%, *P*=1), and the prevalence of other extrahepatic autoimmune diseases also did not significantly differ (*P* > 0.05).

## 4. Discussion

The phenomenon of AIH with elevated serum IgG4 levels has received little attention in clinical practice, and the possible mechanisms have received even less attention. In our study, compared with AIH patients with normal levels, AIH patients with elevated serum IgG4 levels were found to have the clinical characteristics of a higher proportion of male patients, an older age at onset, a high prevalence of cirrhosis, and a low prevalence of EAD at the time of diagnosis. Further logistic regression analysis showed that elevated serum IgG4 levels and male sex were independent risk factors for AIH cirrhosis. In addition, we also detected TH17-related cytokines in some patients and found that serum IL-17 and IL-22 levels were significantly increased in AIH patients with elevated serum IgG4 levels, and these results were also supported by immunohistochemical evaluation of liver histology.

AIH is a common disease in females. The latest practice guidelines of the American Association for the Study of Liver Disease [14] indicate that female AIH patients could account for 71–95% of AIH patients. Interestingly, the proportion of males was higher among the AIH patients with elevated serum IgG4 levels included in our study. Further analysis concluded that elevated serum IgG4 levels and male sex were risk factors for AIH cirrhosis, suggesting that the possible reasons were that male susceptibility genes or abnormal male hormone and elevated serum IgG4 levels were involved in the regulation of the progression of AIH cirrhosis. A study by Czaja and Donaldson [15] concluded that the HLA DR4 frequency in male patients with AIH was less than that in female patients with AIH and that sex can affect type 1 AIH susceptibility and clinical manifestations. Although their study concluded that the liver cirrhosis prevalence rates of men and women at the diagnosis of AIH were not different, our study found that male AIH patients had higher cirrhosis and serum IgG4 levels than female AIH patients. Further analysis revealed that male sex was a risk factor for elevated serum IgG4 levels in AIH. Studies have reported [16, 17] that testosterone has an inhibitory effect on immune cells, for example, inhibiting the production of IgG by B cells [18], while estrogen has an enhancing effect on humoral immunity. Since IgG4 is a subtype of IgG, we speculated that AIH patients with elevated serum IgG4 levels may have significantly decreased androgen levels, which would increase the production of serum IgG4. Moreover, our study concluded that the IgG levels in AIH patients with elevated serum IgG4 levels were higher than those in AIH patients with normal levels, which also supported the speculation on decreased androgen levels in such patients. In our study, regardless of whether male patients or patients with elevated IgG4 levels had a high prevalence of cirrhosis at the time of AIH diagnosis, in general, cirrhosis could reduce the inactivation of estrogen and lead to a relative decrease in androgen levels. Therefore, sex hormone imbalance may be involved in the development of cirrhosis, and elevated serum IgG4 levels may be a marker of sex hormone imbalance in AIH patients. In addition, similar to the results of other studies [11, 19], we found that male AIH patients had a high incidence of cirrhosis at the time of diagnosis, and we also concluded that male sex is a risk factor for AIH cirrhosis. Additionally, no significant difference in the time interval from onset to diagnosis was observed between male and female patients in our study; therefore, the role of sex differences in the development of AIH cirrhosis could not be excluded. Classic AIH is an autoimmune disease that tends to occur in women; however, the proportion of male AIH patients with elevated serum IgG4 levels was increased, and the onset age was older, so this type of patient may represent a separate type of AIH or AIH may even develop into IgG4-RD in the future.

In terms of laboratory examinations, due to the high prevalence of cirrhosis in the group with elevated serum IgG4 levels, the synthesis function of hepatocytes was weakened; therefore, the ALB levels in this group were lower than those in the normal IgG4 group (*P*=0.028), and the PT also showed a trend toward extension. In this study, the rate of liver cirrhosis diagnosed by liver histology was lower than that determined by imaging examinations combined with liver function, coagulation, and platelet measurements. Additionally, liver biopsy can have a 25% false-negative rate [20]; therefore, the diagnosis of liver cirrhosis needs to include a comprehensive evaluation of the liver biopsy, imaging, and serology results. In terms of treatment response, no difference was found between the two groups, indicating that the serum IgG4 level did not affect the immunosuppressive treatment response of AIH. In terms of EAD, our study found that the prevalence of EAD in the elevated IgG4 group was lower than that in the normal group (9.76% vs. 27.03%, *P*=0.023), but the prevalence of liver cirrhosis was higher in the elevated IgG4 group (*P*=0.026). These results are similar to those of Mendes et al. [21] who found that PSC patients with elevated serum IgG4 levels had a lower prevalence of inflammatory bowel disease (*P* < 0.0001) and shorter survival time without liver transplantation (*P*=0.0009). Therefore, similarly, the prognosis of AIH patients with elevated serum IgG4 levels may be worse, because cirrhosis is a poor prognostic factor for AIH [19]. And our study concluded that EAD is a protective factor for preventing IgG4 level elevation; therefore, we speculate that the pathogenesis of cirrhosis in the elevated serum IgG4 group might be different from that in the normal IgG4 group. All IgG4-RD and IgG4-associated AIH patients were excluded from our study. In terms of the correlation between serum IgG4 levels and the amount of IgG4-positive plasma cell infiltration in liver tissues, although the elevated IgG4 group was associated with increased serum IgG4 levels, no obvious IgG4-positive plasma cell infiltration was observed in the liver of the two groups. Chung et al. [22] also reported that serum IgG4 levels were not correlated with IgG4-positive plasma cells in the liver for a subtype of AIH called IgG4-related AIH. However, Umemura et al. [23] suggested that elevated serum IgG4 levels were significantly correlated with the number of IgG4-positive plasma cells in liver tissues in patients with autoimmune pancreatitis with liver involvement. The diagnoses of patients in the above two studies were different, and the sample size was small; therefore, larger sample size is needed in the future to further determine the relationship between serum IgG4 levels and IgG4-positive plasma cell infiltration in the liver of AIH patients. In addition, AIH may be associated with bile duct injury [24]. In our study, the elevated IgG4 group also showed bile duct injury but had no difference in parameters from the normal IgG4 group, although TB, ALP, GGT, and PT showed trends of higher levels in the elevated IgG4 group.

**Figure 3:**
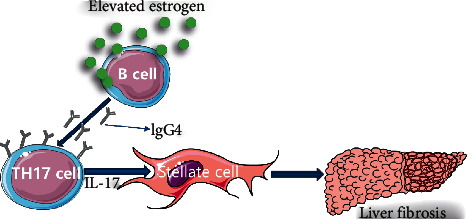
Possible liver fibrosis or cirrhosis pathogenesis of AIH with elevated serum IgG4It has reported IgG4 can target the PD-1 receptor on the surface of T cells to block the PD-1 pathway-mediated inhibition of T cell proliferation and cytokine production [25]. In our study, we speculate AIH with elevated serum IgG4 possibly have relatively elevated estrogen levels that lead to increased IgG4 antibody production by B cells and then IgG4 bind to the surface of TH17 cells to block the PD-1 pathway-mediated inhibition of TH17 cell proliferation and cytokine production. Thus, the proliferation of TH17 cells and the production of related cytokines can be increased to act on hepatic stellate cells which result in a high prevalence of cirrhosis.

A previous study reported [13] that, compared with younger AIH patients, at the time of AIH diagnosis, elderly AIH patients were more often asymptomatic and more frequently positive for ANA; additionally, among the extrahepatic manifestations, AITD were prevalent in the elderly group (25% vs. 3%, *P*=0.02). However, our study found that there was no significant difference in the asymptomatic performance, ANA-positive rate, or remission rate of elderly patients compared with younger patients, which were consistent with a recent Japanese study [25] of 359 AIH patients. Further, similar to many other studies [26, 27], our study found that the prevalence of liver cirrhosis and serum IgG levels was higher in elderly patients than in younger patients, and in general, the prevalence of AITD in extrahepatic autoimmune diseases accounted for the majority. In this study, there was no difference in the age at diagnosis between the elevated serum IgG4 group and the normal group. Furthermore, the age at diagnosis was not included in the equation when the risk factor analysis of cirrhosis was conducted, indicating that age at diagnosis was not related to the occurrence of cirrhosis, which agreed with previous research results [25]. However, the age at onset was older (56 ± 11.43 years old vs. 49.49 ± 13.04 years old, *P*=0.005), and the prevalence of cirrhosis was higher (*P*=0.026) in the elevated serum IgG4 group than in the normal group. One possible reason for these phenomena is that the influence of onset age on the immune response [28] is involved in the cirrhotic pathogenesis of AIH with elevated serum IgG4 levels. An even more likely reason is that elevated serum IgG4 levels are involved in the occurrence of AIH cirrhosis through some mechanism, thus leading to a higher prevalence of cirrhosis in the group with elevated serum IgG4 levels, which is supported by our study concluding that an elevated serum IgG4 level is a risk factor for AIH cirrhosis. However, there is still a lack of knowledge on the role of serum IgG4 in the pathogenesis of AIH and even in IgG4-RD; thus, the specific role of serum IgG4 is not known [29]. Nivolumab is a fully human monoclonal IgG4 antibody targeting the PD-1 receptor that can bind to the PD-1 receptor on the surface of T cells to block the PD-1 pathway-mediated inhibition of T cell proliferation and cytokine production [30]. Many studies have reported that anti-PD-1 treatment is related to the aggravation of autoimmune diseases, such as colitis, vitiligo, and psoriasis [31]. In addition, the PD-1 receptor can be present on the TH17 cell surface [32]. Thus, we speculated that the elevated serum IgG4 levels observed during the pathogenesis of AIH may occur because of IgG4 binding to the PD-1 receptor on the surface of TH17 cells, which could block the PD-1 pathway-mediated inhibition of TH17 cell proliferation and cytokine generation, thereby promoting TH17 cell proliferation and increased production of related cytokines and causing the higher prevalence of cirrhosis observed in AIH patients with elevated serum IgG4 levels. Previous studies reported [6, 7] that the increase in the number of TH17 cells in AIH was associated with the degree of liver fibrosis, and in vitro studies also confirmed that deficiencies in IL-17A and IL-17A receptors could reduce the liver fibrosis caused by CCl4 and bile duct ligation in mice [33]. Other studies [34] also showed that serum IL-17A and IL-22 levels were positively correlated with liver injury in AIH. Therefore, our study detected TH17-related cytokines in some patients, and the results showed that the levels of IL-17A and IL-22 in the elevated IgG4 group were higher than those in the normal IgG4 group. To some extent, this may explain the following: (1) why an elevated serum IgG4 level is a risk factor for AIH cirrhosis and (2) why the rates of cirrhosis and serum IgG level are higher in AIH patients with elevated serum IgG4 levels. Serum IgG4 is probably a marker of severe immune injury and rapid progression of cirrhosis in AIH. In addition, our study also concluded that male sex is a risk factor for AIH cirrhosis. As discussed in the previous paragraph, both AIH patients with elevated serum IgG4 levels and male AIH patients are likely to have relatively low androgen levels and high estrogen levels, and some studies have reported that sex hormones affect the number of TH17 cells and expression of cytokines. For example, in a study on asthma, Fuseini et al. [35] found that the number of TH17 cells and the secretion of IL-17A were increased in the lung tissues of mice with reduced testosterone levels but increased in those with elevated ovarian hormone levels. Testosterone attenuated the IL-17A-mediated inflammatory response by acting on the androgen receptor. In addition, estrogen could increase the production of IL-17A in TH17 cells by acting on the estrogen receptor ER*α* signaling pathway [36]. Therefore, TH17 cells are closely related to the immune disorder and development of cirrhosis in AIH patients with elevated serum IgG4 levels. Moreover, for the treatment of AIH that has progressed to hepatic fibrosis, it may not be sufficient to pursue the therapeutic goal of CR because a study has reported [11] that AIH patients with mild liver fibrosis can progress to cirrhosis within 4 years despite immunosuppressive therapy administration. In the future, more attention should be paid to antifibrotic therapy for AIH patients with fibrosis, such as drug research on anti-TH17 cell-related targets (liver fibrosis/cirrhosis pathogenesis of AIH with elevated serum IgG4 is shown in Figure 3).

There were some limitations to our study. First, this study was a single-center retrospective study, and the sample size of the elevated serum IgG4 group was small. Second, no additional basic studies on the pathogenesis of AIH with elevated IgG4 levels or the role of TH17 in this disease have been conducted. Finally, this study included only patients who had been tested for serum IgG4 and did not analyze other patients who were not tested for serum IgG4, so there was a certain measurement bias.

In conclusion, the proportion of male patients was increased, and the prevalence of cirrhosis was high in AIH patients with elevated serum IgG4 levels at the time of diagnosis. Elevated serum IgG4 levels and male sex are independent risk factors for AIH cirrhosis, and an elevated serum IgG4 level may be a marker of decreased androgen levels and severe immune impairment in AIH patients. TH17 cells probably play an important role in the pathogenesis of cirrhosis in AIH patients with elevated serum IgG4 levels.

## Figures and Tables

**Table 1:** Comparison of baseline characteristics.

	A group (*N* = 41)	B group (*N* = 111)	*P* value
Male *n* (%)	14 (34.15)	14 (12.61)	**0.002**
Age at onset (years)	**56** **±** **11.43**	**49.49** **±** **13.04**	**0.005**
Age at diagnosis (years)	57.32 ± 11.54	52.90 ± 12.64	**0.052**
Pretreatment IAIHG score	15 (13–18.5)	17 (13–19)	0.324
Simplified diagnostic criteria score^A^	7 (6–8)	7 (6–8)	0.511
Cirrhosis *n* (%)	**23 (56.10)**	**40 (36.04)**	**0.026**
Platelet × 10^3^/L (NR 100-300)	129 (88–168)	123 (85–186)	0.977
PT (NR 9.6–12.8)	13.3 (1235–16.65)	12.95 (11.80–14.50)	0.063
ALT (IU/L) (NR < 40)	110 (36–293)	114 (58–270)	0.544
AST (IU/L) (NR < 35)	128 (60–321)	134 (65–283)	0.692
TB (umol/L) (NR 5–28)	38.5 (20.25–91.05)	26.5 (17.3–66.4)	0.078
ALP (IU/L) (NR 50- 135)	149 (112–201)	134 (93–172)	0.056
GGT (IU/L) (NR < 45)	137 (61.5–222)	92 (43–173)	0.078
ALB (g/L) (NR 40–55)	34.4 (27.75–43.5)	38.6 (33.1–42.5)	**0.028**
GLB (g/L) (NR 20–40)	42.1 (34.05–47.75)	37 (31.1–43.7)	**0.043**
IgG (g/L) (NR 8–15.5)	24.8 (21.9–31.7)	20.9 (16.9–28.6)	**0.016**
IgM (mg/L) (NR 700–2200)	1400 (968.5–2005)	1775 (1220–2460)	**0.013**
IgG4 (g/L) (NR ≤ 1.35)	2.84 (1.82–3.5)	0.47 (0.262–0.837)	**0.000**
ANA positivity *n* (%)	27 (5.85)	86 (77.48)	0.145
Anti-LKM1 positivity *n* (%)	1 (2.44)	2 (1.82)	1.000
Anti-LC1 positivity *n* (%)	1 (2.44)	0 (0)	0.270
Anti-SLA/LP positivity *n* (%)	2 (4.88)	15 (13.51	0.227
p-ANCA positivity *n* (%)	1 (2.44)	12 (10.81)	0.190
EAD *n* (%)	4 (9.76)	30 (27.03)	**0.023**
Histology (severity of inflammation)^B^	***N*** **=** **39**	***N*** **=** **107**	—
G1 *n* (%)	4 (10.26)	10 (9.35)	1.000
G2 *n* (%)	8 (20.51)	29 (27.10)	0.418
G3 *n* (%)	27 (69.23)	61 (57.01)	0.182
G4 *n* (%)	0 (0)	7 (6.54)	0.230
Fibrosis stages at diagnosis	***N*** **=** **39**	***N*** **=** **107**	—
S0 *n* (%)	1 (2.56)	4 (3.73)	1.000
S1 *n* (%)	7 (17.95)	28 (26.17)	0.303
S2 *n* (%)	6 (15.38)	32 (29.90)	0.077
S3 *n* (%)	11 (28.10)	23 (21.50)	0.396
S4 *n* (%)	14 (35.90)	20 (18.69)	**0.030**
Bile lesion *n* (%)	6 (15.38)	25 (23.36)	0.613
Remission^C^*n* (%)	23 (69.70%)	67 (76.14%)	0.470

PT, prothrombin time; ALT, alanine aminotransferase; AST, aspartate aminotransferase; TB, total bilirubin; ALP, alkaline phosphatase; GGT, gamma-glutamyl transferase; ALB, albumin; GLB, globulin; IgG, immunoglobulin G; IgM, immunoglobulin M; IgG4, immunoglobulin G4; ANA, anti-nuclear antibody; anti-LKM1, antibodies to liver kidney microsome type 1; anti-LC1, antibodies to liver cytosol type 1; anti-SLA/LP, antisoluble liver antigen/liver pancreas antibody; p-ANCA, perinuclear antineutrophil cytoplasmic antibody; and EAD, extrahepatic autoimmune diseases. A: simplified diagnostic scoring system of the IAIHG [12]. B: 6 patients who did not receive liver biopsies were excluded. The liver biopsy results of all patients had inflammation presentation; thus, there was no G0 grade for inflammation grade. C: there were 33 patients in group A and 88 patients in group B who received regular treatment with a course of treatment longer than 6 months.

**Table 2:** Comparison of the age at onset, IgG4 level, cirrhosis prevalence, and other parameters between male and female patients.

	Male (*N* = 28)	Female (*N* = 124)	*P* value
Age at onset (years)	53.64 ± 15.998	50.7 ± 12.129	0.366
IgG4 g/L (NR ≤ 1.35)	1.37 (0.689–2.772)	0.676 (0.298–1.150)	**0.002**
IgG g/L (NR 8–15.5)	23.1 (18.075–26.8)	22.8 (17.375–30.975)	0.991
Cirrhosis *n* (%)	18 (64.29)	45 (36.29)	**0.007**
Time^†^ (year)	1 (1–3)	1.5 (1–3.75)	0.688
Histology^‡^ (severity of inflammation)	*N* = 28	*N* = 118	—
G1 *n* (%)	3 (10.71)	11 (9.32)	1.000
G2 *n* (%)	6 (21.43)	31 (26.27)	0.596
G3 *n* (%)	18 (64.29)	69 (58.47)	0.573
G4 *n* (%)	1 (3.57)	6 (5.08)	1.000
Fibrosis stages at diagnosis	*N* = 28	*N* = 118	—
S0 *n* (%)	2 (7.14)	3 (2.54)	0.244
S1 *n* (%)	4 (14.28)	31 (26.27)	0.182
S2 *n* (%)	6 (21.43)	32 (27.12)	0.537
S3 *n* (%)	6 (21.43)	28 (23.73)	0.796
S4 *n* (%)	10 (35.71)	24 (20.34)	**0.084**

IgG, immunoglobulin G; IgG4, immunoglobulin G4. †The time interval between the onset time and the diagnosis time. ‡6 patients in the female group who did not receive liver biopsies were excluded.

**Table 3:** Risk factor analysis of serum IgG4 level elevation.

	OR	95% CI	SE	*P* value
Male	**2.809**	1.034–7.629	0.510	**0.043**
EAD	**0.274**	0.078–0.967	0.643	**0.044**
Age at onset (years)	1.455	1.122–1.886	0.132	0.005
Age at diagnosis (years)	0.709	0.548–0.918	0.131	0.009

EAD, extrahepatic autoimmune diseases; OR, odds ratio; and CI, confidence interval; SE, standard error.

**Table 4:** Risk factor analysis of AIH cirrhosis.

	OR	95% CI	SE	*P* value
Male	**4.293**	**1.592–11.575**	0.506	**0.004**
EAD	1.474	0.579–5.748	0.476	0.416
IgG4	**2.566**	**1.065–6.187**	0.449	**0.036**
TB	1.001	0.998–1.00	0.002	0.461
ALP	1.010	1.003–1.017	0.004	0.005
GGT	0.994	0.991–0.998	0.002	0.002
IgM	1.000	1.000–1.001	0.000	0.006

EAD, extrahepatic autoimmune diseases; IgG4, immunoglobulin G4; TB, total bilirubin; ALP, alkaline phosphatase; GGT, gamma-glutamyl transferase; IgM, immunoglobulin M; OR, odds ratio; SE, standard error; and CI, confidence interval.

**Table 5:** Comparison of TH17 cytokine levels between the elevated serum IgG4 group and the normal group.

	A group (*N* = 15)	B group (*N* = 53)	*P* value
IL-17A (pg/ml)	2.85 (2.16–3.73)	2.16 (1.29–2.85)	**0.017**
IL-21 (pg/ml)	8.67 (7.08–9.77)	8.26 (7.08–10.12)	0.705
IL-22 (pg/ml)	7.39 (3.38–10.40)	4.69 (2.90–6.58)	**0.042**

IL-17, interleukin 17; IL-21, interleukin 21; and IL-22, interleukin 22.

**Table 6:** Comparison of baseline information between elderly and younger patients.

	Younger group (*N* = 117) (<65 years)	Elderly group (*N* = 35) (≥65 years)	*P* value
Male *n* (%)	19 (16.24)	9 (25.71)	0.205
Cirrhosis *n* (%)	43 **(36.75)**	20 **(57.14)**	**0.032**
Asymptomatic presentation	39 (33.33)	11 (31.43)	**0.833**
PT (NR 9.6–12.8)	13. (11.9–150	13.4 (12.2–15.7)	0.359
ALT (IU/L) (NR < 40)	134 (57–303)	87 (38–164)	0.09
AST (IU/L) (NR < 35)	134 (61–292)	128 (69–299)	0.759
TB (umol/L) (NR 5–28)	30.6 (17.75–72.30)	30.9 (17.7–80.0)	0.738
ALP (IU/L) (NR 50- 135)	137 (94.5–175)	142 (103–174)	0.609
GGT (IU/L) (NR < 45)	96 (48–183)	136 (48–255)	0.243
ALB (g/L) (NR 40–55)	38.5 (32.25–42.85)	34.9 (29.9–41.7)	0.133
IgG (g/L) (NR 8–15.5)	**22 (16.7–28)**	**24.8 (20.4–32.4)**	**0.017**
IgG4 (g/L) (NR ≤ 1.35)	0.728 (0.348–1.16)	0.918 (0.338–2.84)	0.297
ANA positivity *n* (%)	85 (72.65)	28 (80.0)	0.382
Anti-LKM1 positivity *n* (%)	2 (1.71)	1 (2.86)	0.547
Anti-LC1 positivity *n* (%)	0 (0.00)	1 (2.86)	0.230
Anti-SLA/LP positivity *n* (%)	12 (12.82)	2 (5.71)	0.387
EAD *n* (%)	28 (23.93)	6 (17.1)	**0.398**
Remission^†^*n* (%)	71 (73.20)	19 (79.17)	0.549

PT, prothrombin time; ALT, alanine aminotransferase; AST, aspartate aminotransferase; TB, total bilirubin; ALP, alkaline phosphatase; GGT, gamma-glutamyl transferase; ALB, albumin; IgG4, immunoglobulin G4; ANA, anti-nuclear antibody; anti-LKM1, antibodies to liver kidney microsome type 1; anti-LC1, antibodies to liver cytosol type 1; anti-SLA/LP, antisoluble liver antigen/liver pancreas antibody; and EAD, extrahepatic autoimmune diseases. † There were 24 patients in the elderly group and 97 patients in the younger group who received regular treatment with a course of treatment longer than 6 months, respectively.

**Table 7:** Distribution of extrahepatic autoimmune diseases in our study at AIH diagnosis.

	All patients (19–84 years) (*N* = 152) (%)	Elderly (≥65 years) (*N* = 35) (%)	Younger (<65 years) (*N* = 117) (%)
Total EAD	37 (23.34)	6 (17.14)	34 (29.06)
AITD	13 (8.55)	3 (8.57)	10 (8.55)
SS	12 (7.89)	1 (2.86)	12 (10.26)
RA	3 (1.97)	—	3 (2.56)
SLE	2 (1.32)	—	2 (1.71)
CTD	1 (0.66)	—	1 (0.85)
ITP	1 (0.66)	—	1 (0.85)
Vitiligo	1 (0.66)	—	1 (0.85)
Psoriasis	2 (1.32)	—	2 (1.71)
Hemolytic anemia	1 (0.66)	1 (2.86)	1 (0.85)
Erythema nodosa	1 (0.66)	1 (2.86)	1 (0.85)

EAD, extrahepatic autoimmune diseases; AITD, autoimmune thyroid disease; SS, Sjögren's syndrome; RA, rheumatoid arthritis; SLE, systemic lupus erythematosus; CTD, connective tissues disease; and ITP, idiopathic thrombocytopenic purpura.

## Data Availability

Readers can access the data supporting the conclusions of the study upon request to the corresponding author (e-mail: yangli_hx@scu.edu.cn).
